# The weaker sex: Male lingcod (*Ophiodon elongatus*) with blue color polymorphism are more burdened by parasites than are other sex–color combinations

**DOI:** 10.1371/journal.pone.0261202

**Published:** 2021-12-31

**Authors:** Chelsea L. Wood, Katie L. Leslie, Alanna Greene, Laurel S. Lam, Bonnie Basnett, Scott L. Hamilton, Jameal F. Samhouri

**Affiliations:** 1 School of Aquatic and Fishery Sciences, University of Washington, Seattle, WA, United States of America; 2 School of Marine and Environmental Affairs, University of Washington, Seattle, WA, United States of America; 3 Pacific States Marine Fisheries Commission, Northwest Fisheries Science Center, National Marine Fisheries Service, National Oceanic and Atmospheric Administration, Seattle, WA, United States of America; 4 Moss Landing Marine Laboratories, San Jose State University, Moss Landing, CA, United States of America; 5 Conservation Biology Division, Northwest Fisheries Science Center, National Marine Fisheries Service, National Oceanic & Atmospheric Administration, Seattle, WA, United States of America; University of Pretoria, SOUTH AFRICA

## Abstract

The unusual blue color polymorphism of lingcod (*Ophiodon elongatus*) is the subject of much speculation but little empirical research; ~20% of lingcod individuals exhibit this striking blue color morph, which is discrete from and found within the same populations as the more common brown morph. In other species, color polymorphisms are intimately linked with host–parasite interactions, which led us to ask whether blue coloration in lingcod might be associated with parasitism, either as cause or effect. To test how color and parasitism are related in this host species, we performed parasitological dissection of 89 lingcod individuals collected across more than 26 degrees of latitude from Alaska, Washington, and California, USA. We found that male lingcod carried 1.89 times more parasites if they were blue than if they were brown, whereas there was no difference in parasite burden between blue and brown female lingcod. Blue individuals of both sexes had lower hepatosomatic index (i.e., relative liver weight) values than did brown individuals, indicating that blueness is associated with poor body condition. The immune systems of male vertebrates are typically less effective than those of females, due to the immunocompromising properties of male sex hormones; this might explain why blueness is associated with elevated parasite burdens in males but not in females. What remains to be determined is whether parasites induce physiological damage that produces blueness or if both blue coloration and parasite burden are driven by some unmeasured variable, such as starvation. Although our study cannot discriminate between these possibilities, our data suggest that the immune system could be involved in the blue color polymorphism–an exciting jumping-off point for future research to definitively identify the cause of lingcod blueness and a hint that immunocompetence and parasitism may play a role in lingcod population dynamics.

## Introduction

In a textbook example of how the environment can shape expression of a color polymorphism, the selective landscape of the peppered moth (*Biston betularia*) shifted during the Industrial Revolution, such that dark-colored morphs that could camouflage against soot-blackened trees became favored over the light-colored morphs that had previously been dominant [1]. This “industrial melanism” has long been believed to protect dark-colored moths against visual predation by birds [2]. However, recent research suggests an alternative explanation for the shift from light-colored to dark-colored morphs: the chemical precursors of the melanin that darkens moth wings also give rise to components of the immune system [3]. Therefore, dark-colored morphs might be better protected against the parasitic threats of the industrial age, and this famous color polymorphism may be maintained by parasitism, not predation.

This example is not unique, as color and parasitism are intimately linked across animal taxa through a variety of mechanisms [4, 5]; taxa in which such linkages have been documented include birds [e.g., 6, 7], amphibians [e.g., 8], and insects [e.g., 3, 9]. For example, bright coloration in male birds is linked to infection pressure, with coloration serving as an “honest signal” of resistance to parasites among males competing for mating opportunities [4]. Color may also be linked to immunity via pleiotropy, as in birds [e.g., 7, 10] or moths [e.g., 3] whose genes for melanogenesis also regulate immune function. Pathological effects of parasitism can cause incidental changes in host coloration; for example, when it is infected by the trematode parasite *Cryptocotyle lingua*, the common periwinkle (*Littorina littorea*) exhibits a dark orange coloration, which might arise because the trematode destroys the digestive tissues of the snail, releasing carotenoids that are deposited in the tissues of the foot [11]. Color polymorphisms are therefore better understood with information on the parasitic pressures faced by the polymorphic host.

**Fig 1:**
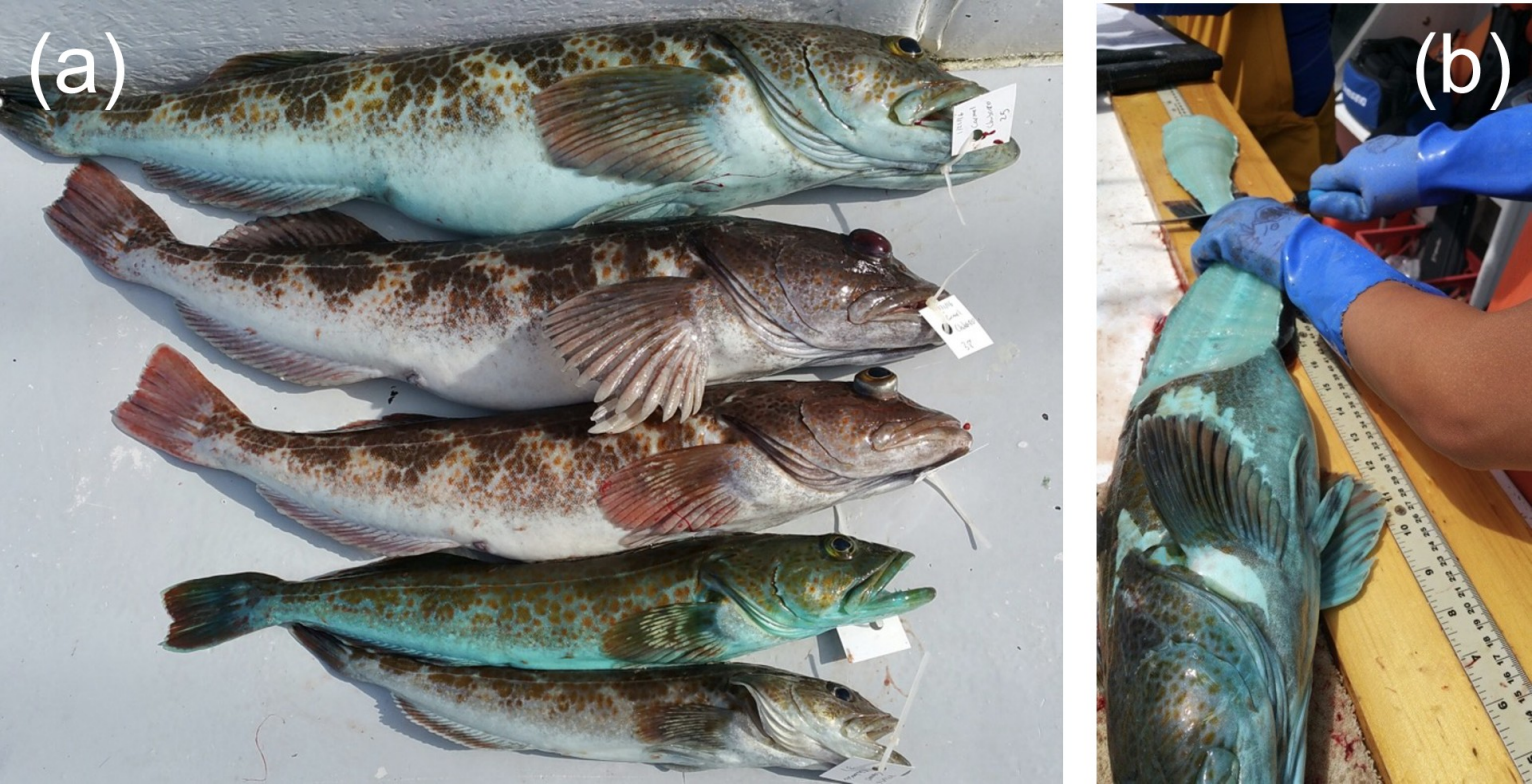
(a) Lingcod (*Ophiodon elongatus*) of two color morphs: blue (topmost and fourth-from-top) and brown (second-from-top, third-from-top, and bottommost). (b) Blue coloration affects both external and internal tissues. Courtesy of Laurel Lam / NOAA.

Although color polymorphisms are common, few natural history oddities can match the blue color morph of lingcod (*Ophiodon elongatus*) for whimsy (**Fig 1**). Despite the fact that ~20% of lingcod individuals on the west coast of North America exhibit this striking blue color morph (where both external and internal tissues are blue), which is discrete from and found within the same populations as the more common brown morph (where external color is brown and musculature is white), science has yet to explain why [12]. Similar blue coloration is observed in other members of the greenlings (Family Hexagrammidae), as well as in numerous species of sculpins (Family Cottidae) [12]. Among the sculpins, blueness arises from the bile pigment biliverdin, which circulates in lymph, suffuses tissues, and causes blue coloration in the skin and musculature [13–15]; biliverdin has long been assumed to drive blue coloration in lingcod as well [12], although this hypothesis remains to be tested. A product of heme catabolism, biliverdin gives the gallbladder and the bile within their characteristic blue-green color and dysfunction of the liver or gallbladder can result in this material suffusing tissues [16]. For example, in other fish species, blueness due to elevated levels of biliverdin is linked to starvation [17]. Blue coloration in lingcod has long been anecdotally attributed to individual variation in dietary preferences, with blue individuals posited to prefer prey items that trigger biliverdin release or that have high levels of biliverdin in their tissues [18]. However, it is as yet unclear, for any fish species, whether the blue color polymorphism is environmentally determined, has a genetic component, or both.

We became curious about the role of parasitism in lingcod blueness for a few reasons. Having performed parasitological dissection of a number of (non-blue) lingcod at the outset of this project, two of us noted that this species has among the highest average burdens of metazoan parasites that we have ever encountered in dissections of dozens of fish species (CL Wood and KL Leslie, personal observation). Furthermore, a parallel study uncovered differences in fatty acid signatures between blue and brown lingcod and found that blue lingcod were, on average, smaller than brown lingcod [18]. Because this finding suggested an association between blueness and diet, we wondered whether blueness might, by extension, also be associated with parasitism. A large proportion of lingcod parasites are transmitted from prey to lingcod predators (CL Wood and KL Leslie, personal observation), so it stood to reason that some trophically transmitted parasites could cause blueness or that the diet differences that drove blueness might also drive differences in parasite burden (e.g., low body condition due to starvation reduces resistance to parasites). Galloway et al. [18] also found that a greater proportion of blue lingcod were female, compared to brown lingcod, and that blue individuals possessed lower concentrations of ω-6 fatty acids–precursor molecules for anti-inflammatory eicosanoids. In general, male vertebrates tend to be more susceptible to parasitic infection than are females [19, 20]; male lingcod undergo periods of starvation during the nest-guarding period [21], and may therefore be particularly susceptibility to infection. If coloration is associated with parasite burden, we might expect a greater difference in burden between blue and brown individuals for male lingcod compared to female lingcod. We therefore designed our study to test the associations among coloration, sex, and body condition. We lay out some scenarios by which these variables may be linked below. Note that we provide these explanations as background only; our study is observational and we can draw no conclusions as to the causal linkages among coloration, sex, and body condition. However, the correlations we can uncover may point future researchers toward productive experiments that can explicitly test the mechanisms laid out below.

Non-blue (hereafter, “brown”) lingcod might be more heavily burdened by parasites than blue lingcod for several reasons. If blueness in lingcod is an “honest signal” of resistance (i.e., the ability to prevent successful parasitic infection after exposure) against parasites (sensu [4]), then the sex under selection (presumably males) should exhibit blue coloration while the choosy sex (presumably females) does not, and blue male lingcod should have lower burdens than brown male lingcod. It is also possible that blue coloration may reflect the production of an antiparasitic compound that increases host resistance to infection; for example, immunocompetence varies between darker and lighter morphs of various bird species, although the direction of this effect (i.e., whether greater melanization increases or decreases immunocompetence) varies among species [22]. If blue coloration is an adaptation to increase resistance to infection, then we would predict that parasites that elicit a strong immune response (e.g., see Appendix M in [23]) would be more likely to occur in brown than in blue lingcod. For example, endoparasites may trigger a stronger immune response than do ectoparasites [24], so we anticipated that, if color were linked to immunity, these two groups of parasites might differ in their response to host coloration (e.g., lower endoparasite abundance in blue individuals than brown, but equal numbers of ectoparasites).

Alternatively, blue lingcod might be more heavily burdened by parasites than brown lingcod. This could happen if blueness is an honest signal of tolerance to parasites. Tolerance refers to the ability of hosts to physiologically limit the fitness impacts of parasitism, and is distinct from resistance, or the ability to prevent parasitic infection in the first place [25]. The androgens that produce secondary sex characteristics in male vertebrates erode immune function, increasing susceptibility to infection [26–28]. On top of this endocrine disadvantage, male lingcod may be further immunocompromised by periods of starvation during the nest-guarding period [21]. Therefore, in contrast with the prediction that high-quality males displaying “showy” traits should have lower parasite burdens, the “immunocompetence handicap hypothesis” predicts that showy males should have higher parasite burdens than males without showy traits, and that the honest signal indicated by the showy trait is the ability to tolerate parasitic infection rather than to resist it [29]. If blue coloration in lingcod were a sexually selected trait linked to androgens and signaled parasite tolerance (rather than resistance), we would predict that blue coloration would occur primarily in males, and that there would be greater parasite burdens among blue males compared to brown males.

Blue lingcod might also carry heavier parasite burdens than brown individuals if blue coloration is a collateral impact of parasitism, resulting from the disruption of some organ system by infection. The liver or gallbladder are likely candidates, given that the biliverdin to which blue coloration is attributed is a bile pigment [13–15]. In this case, we would predict greater parasite burdens among blue individuals compared to brown individuals, driven primarily by the few parasite species whose specific mechanisms of physiological disruption (e.g., of the liver or gallbladder) produce the pigments leading to blue coloration. We would only expect a difference between males and females in the frequency of the blue trait or in the parasite burden of blue versus brown individuals if one sex were more prone to parasite-induced organ damage; the sex that is more susceptible to organ damage would have a higher frequency of the blue trait overall, and the difference in parasite burden of blue versus brown individuals would be lower for the sex that is more susceptible to organ damage compared to other sex.

Similarly, behavioral differences among individuals might both mediate their exposure to agents that control coloration and their exposure to parasites. For example, some lingcod individuals might have dietary preferences for prey items that trigger biliverdin release or that have high levels of biliverdin in their tissues. If these prey items also carry high parasite burdens, then blue coloration and parasite burden could be positively correlated. In this case, we would only expect a difference between males and females in the frequency of the blue trait or in the parasite burden of blue versus brown individuals if one sex had a stronger dietary preference than the other for biliverdin-associated prey.

Finally, perhaps body condition both induces the biliverdin release linked to blue coloration and simultaneously erodes immune defenses against parasitic infection [30]. In other fish species, starvation can induce blueness [17]. Starvation may also erode immune defenses, resulting in a greater burden of infection across parasite taxa [31–35]. In this scenario, we might also expect a greater burden of parasites among blue males than among blue females, given males’ inherent, pre-existing susceptibility to infection [19, 20]. Alternatively, if there are sex differences in body condition, this could lead to different frequencies of blueness between males and females, as well as lower resistance and therefore greater parasite abundance for the sex with lower body condition.

Given the rich variety of mechanisms that might link sex, coloration, and parasite burden, we were interested in assessing the associations among these variables. To that end, we sampled lingcod across more than 26 degrees of latitude from Alaska, Washington, and California, USA, and performed parasitological dissections on 89 lingcod individuals, representing a range of sizes, sexes, colors, depths of collection, and geographic locations.

## Materials and methods

We sought to build on the findings of Galloway et al. [18] by creating a complementary dataset on parasite burden for the same suite of fishes that had been analyzed in that study. We used a subset of the fishes collected for Galloway et al. [18] to create that parasitological dataset.

### Lingcod collection

**Fig 2:**
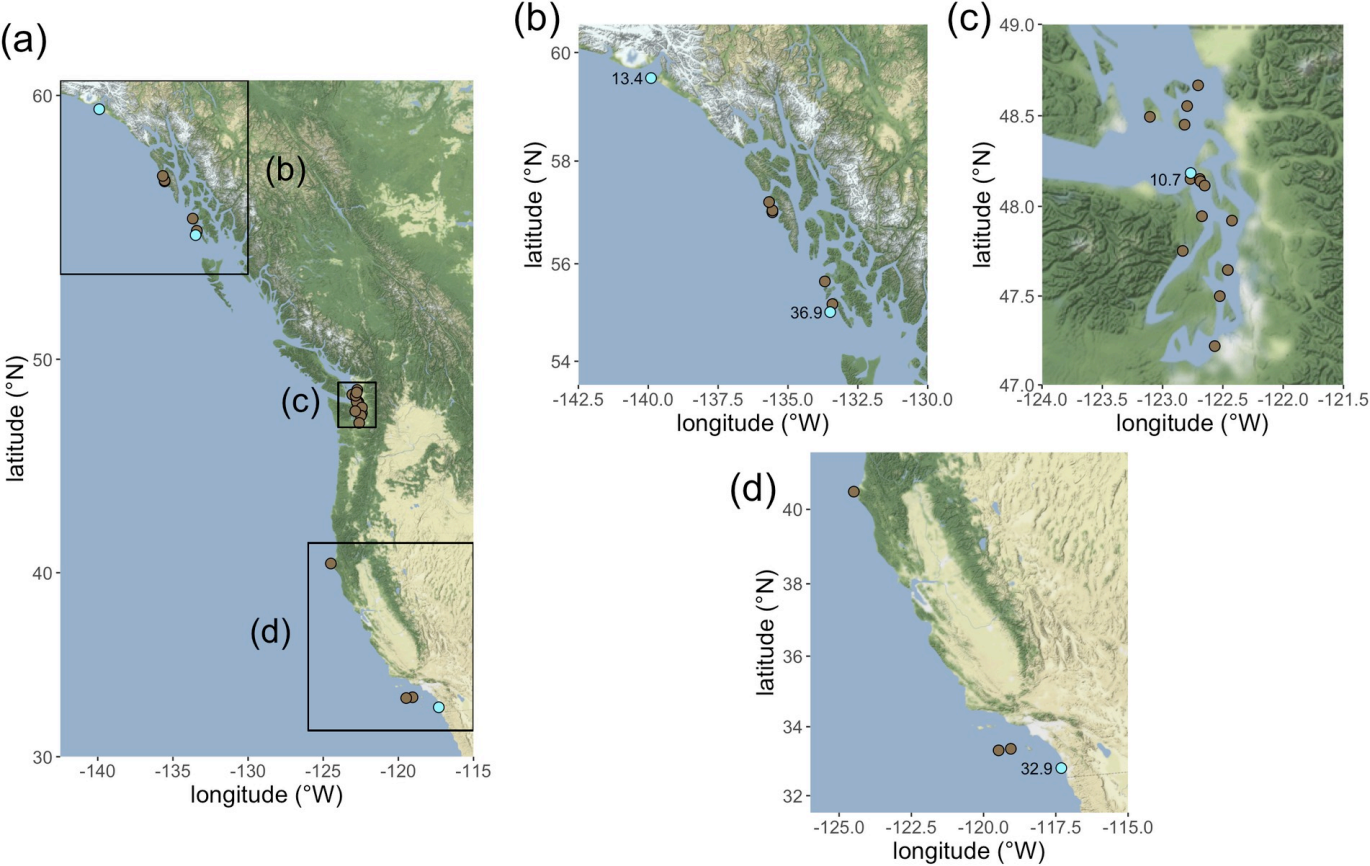
Map of study region with sampling sites indicated. Sites where any blue male (total n = 4) was collected are indicated in blue; sites that yielded only brown males are indicated in brown. The depth of collection of each blue male is noted (in meters). (a) Full study extent with insets displaying the boundaries of maps (b), (c), and (c). Sampling sites in (b) Alaska, (c) Puget Sound, Washington, (d) northern and southern California. Site locations in Puget Sound are jittered to allow better visualization of nearby sites.

Lingcod (n = 2,251) were collected between 2015 and 2017 using hook-and-line methods from chartered commercial passenger fishing vessels (CPFVs) from Yakutat, Alaska to San Diego, California, USA (54°30’N–34°30’N). The following seven regions were broadly identified: Southeast Alaska (54°30’N–59°48’N), Puget Sound and coastal Washington (46°16’N–49°N), Oregon (42°N–46°16’N), northern California (38°02’N–42°N), central California (34°30’N–38°02’N), and southern California (32°32’N–34°30’N; **Fig 2**). Note that samples for parasitological analysis were drawn from only four of these seven regions, spanning most of the geographic range: Southeast Alaska, Washington, northern California, and southern California. Three to four fishing ports were selected per region (n = 24 ports total) based on location and accessibility of CPFVs. To ensure representation of lingcod across a wide range of size and age classes, we sampled shallow (<200 ft) and deep (200–550 ft) nearshore and offshore rocky reefs equally. On average, we fished for 2.5 days out of each port at different locations (shallow and deep) to obtain the targeted sample size of 75–100 lingcod per port. Additional samples were provided by the Alaska commercial longline fishery, the NWFSC Rockfish Bycatch Study in Puget Sound [36], the Oregon Department of Fish and Wildlife Marine Reserves Program [37], and the California Collaborative Fisheries Research Program [38]. With the exception of lingcod collected by the Alaska commercial longline fishery, sampling design from all other lingcod collection methods are similar to that of the current study, with respect to chartering CPFVs for single day trips, using volunteer anglers, and targeting nearshore fishing grounds.

We measured several phenotypic traits for each of the lingcod included in the coastwide dataset. Color was recorded as blue or brown immediately upon capture, then reassessed in the lab (as chromatophores on fish skin are known to undergo apoptosis, causing external color to change upon death) [39]. Color was determined internally based on muscle tissue and fin membrane coloration, as well as externally based on skin color. There was an observed gradient in “blueness” (**Fig 1A**), but color was noted as binary (brown or blue). If there was disagreement between internal and external coloration, we used the internal color, as it may represent a stronger phenotypic expression of the blue trait. To our knowledge, there is no scholarship on the process of post-mortem color degradation in lingcod, but we do know that internal blue coloration persists after death, because blue filets are sold in seafood markets and fetch a higher price than the more typical white filets; in fact, among seafood processors, it is known that blue coloration will remain in blue filets until cooking [18]. For this reason, we use internal coloration to demarcate blue from brown individuals. Sex was determined externally (based on the presence or absence of a conical papilla) and internally (based on the presence of ovaries or testes). Total length was measured to the nearest 0.1 cm and total mass was measured to the nearest 0.01 kg. Internal organs were removed so that gonad mass could be measured to the nearest 0.0001 kg and liver mass to the nearest 0.0001 kg.

### Parasitological dissection of lingcod

Within the coastwide group of lingcod, we sub-sampled 89 for parasitological study. These individuals were immediately measured and bagged upon capture to retain external parasites, which can otherwise drop off their host after death. For each of the 89 fish selected for parasitological dissection, we performed a comprehensive parasitological examination designed to detect most metazoan parasites. Processing time per fish ranged from 4 to 30 hours and averaged 16 hours. We did not search for myxozoan parasites, but all other metazoan parasites should have been detected by our standard parasitological dissection techniques (described in detail in **S1 Text**). We identified all parasite taxa to the lowest possible taxonomic level using keys, host–parasite checklists, regional parasite identification guides, and primary literature.

### Statistical analysis

We tested for differences in the frequency of the blue color morph between male and female lingcod in the full, coastwide dataset with a chi-squared test. This test was conducted merely to better understand the context for the parasitological data; a formal analysis of the differences in frequency of blue fish as a function of sex, depth, and geography appears in Galloway et al. [18].

We used generalized linear mixed models to assess the correlates of parasite burden for the subset of 89 lingcod selected for parasitological analysis. In these models, parasite abundance (number of parasites per fish) was the response variable. The generalized linear mixed-effects model took the form,

$$\begin{array}{l} parasite\_count_{ijkl}∼depth_{j}+sex_{j}+color_{j}+sex_{j}\;*\;color_{j}+\\ \;\left(1|sampling\_region_{l}/sampling\_location_{k}/fish\_ID_{j})+(1+sex_{j}\;*\;color_{j}\right|parasite\_taxon_{i})\\ \;+offset(log(total\_length_{j})), \end{array}$$

where the response variable_*ijk*_ represents the count of the *i*th parasite taxon in the *j*th fish collected from the *k*th site in the *l*th region. Fixed effects included: the depth of collection of the fish, the sex of the fish (male or female), the color of the fish (brown or blue), and the interaction between sex and color. We included fish identification code nested within sampling location nested within sampling region (to account for similarity in parasite burden across parasite taxa within individual fish and among fish collected from the same sampling location or sampling region) as a random intercept. We also included the interaction of sex * color with a random slope and random intercept varying across parasite taxa, to allow calculation of a unique estimate of the interaction effect for each parasite taxon. To account for the fact that larger fish carry more parasites than smaller fish, we included an offset term for log(total length of fish in cm), which effectively converts the response variable to a rate (number of parasites per cm of fish total length). All numeric values were scaled prior to analysis by using the *scale()* function in base R and then adding 2 (to avoid negative values) and back-transformed after analysis to return them to their original units. We checked for collinearity by assessing whether variance inflation factors (VIF) were < 5 [40]. Response variables were modeled as a negative binomial distribution to account for overdispersion using the *glm*.*nb()* function in the lme4 package in R [41].

Realizing that we had very low replication in one cell of our color * sex interaction (n = 4 blue males; **Fig 2**), we sought to test whether the patterns revealed by the analysis above could have arisen due to random chance. We performed a permutation test in two iterations. In the first iteration, we randomly assigned four male fish in our dataset to be “blue” and the remainder to be “brown”. Females were not randomly assigned to a color, but instead retained their actual color assignment in the simulated dataset. This choice was made because the analysis above revealed extreme results for blue males and for no other combination of sex and color; we suspected that this might have been due to the exceptionally low number of blue males, and therefore tested the likelihood of obtaining such an extreme result with an identically sized random sample of males. After randomly appointing four “blue” males from among all the males in the dataset, we performed the analysis above and stored the resulting z-value for the interaction color * sex. We repeated this procedure 500 times and then calculated the proportion of runs in which the actual z-value for the interaction color * sex was more extreme (in either the positive or negative direction) than the simulated z-value for the interaction.

In the second iteration of the permutation test, we again randomly assigned four male fish in our dataset to be “blue” and the remainder to be “brown”. In contrast with the iteration above, however, we then randomly assigned 11 female fish (i.e., the same number as the actual number of blue female fish found in the dataset) to be “blue” and the remainder to be “brown”. This choice was made because we surmised that the extreme results for blue males could have arisen by comparison to blue and brown females, and that random chance could have led to high rates of infection for brown females and low rates of infection for blue females, producing the significant interaction between sex and color. After randomly appointing four “blue” males from among all the males in the dataset and 11 “blue” females from among all the females in the dataset, we performed the analysis above and stored the resulting z-value for the interaction color * sex. We repeated this procedure 500 times and then calculated the proportion of runs in which the actual z-value for the interaction color * sex was more extreme (in either the positive or negative direction) than the simulated z-value for the interaction.

Having investigated the overall patterns of parasite burden across parasite taxa, we then sought to understand whether individual parasite taxa differed in their responses to the interaction of host coloration and sex. From the model above, we extracted the random slope of the sex * color interaction for each parasite taxon and the variance associated with each slope, using the *ranef()* function in the lme4 package in R [41]. We then plotted these random slopes and their standard errors.

To assess the response of parasite community composition to host sex and color, we performed a multivariate analysis. We assessed whether parasite community composition varied among sex–color combinations with a PERMANOVA analysis, using the *adonis()* function in the vegan package in R [42]. The PERMANOVA model included the fixed factors sex–color combination (since *adonis()* does not support interaction terms), sampling location, and sampling depth.

Finally, we performed two post-hoc tests. In light of our results (see below) and because Galloway et al. [18] detected differences in fatty acid signatures between blue and brown individuals, we began to suspect that perhaps differences in diet, and specifically starvation, were driving both blueness and parasite burden. Using the full, coastwide dataset, we tested whether Fulton’s K and the hepatosomatic index of blue individuals differed from those of brown individuals. Fulton’s K condition factor was calculated as mass divided by the cube of length (to account for the fact that weight changes in three dimensions as length changes in one) [43]. High K values signify that a fish is heavier, and potentially healthier, for a given length. We calculated the hepatosomatic index (HSI) as liver mass divided by body mass, a measure of the energy reserve status of a fish, where greater values indicate better condition [43]. HSI was fourth-root transformed to improve normality. The general linear mixed-effects models took the form,

$$\begin{array}{c} response\_variable_{ijk}∼depth_{j}+sex_{j}+color_{j}+sex_{j}\;*\;color_{j}+\\ (1|sampling\_region_{k}/sampling\_location_{j}), \end{array}$$

where the response_variable_*ijk*_ represents either the Fulton’s K or hepatosomatic index of the *i*th fish collected from the *j*th site in the *k*th region. Fixed effects included: the depth of collection of the fish, the sex of the fish (male or female), the color of the fish (brown or blue), and the interaction between sex and color. We included sampling location nested within sampling region (to account for similarity among fish collected from the same sampling location or sampling region) as a random intercept. We excluded one outlier where an impossibly high body mass was accidentally recorded. All numeric predictors were scaled prior to analysis by using the *scale()* function in base R and then adding 2 (to avoid negative values) and back-transformed after analysis to return them to their original units. The model was run using the *glmer()* function in the lme4 package in R [41].

## Results

**Fig 3:**
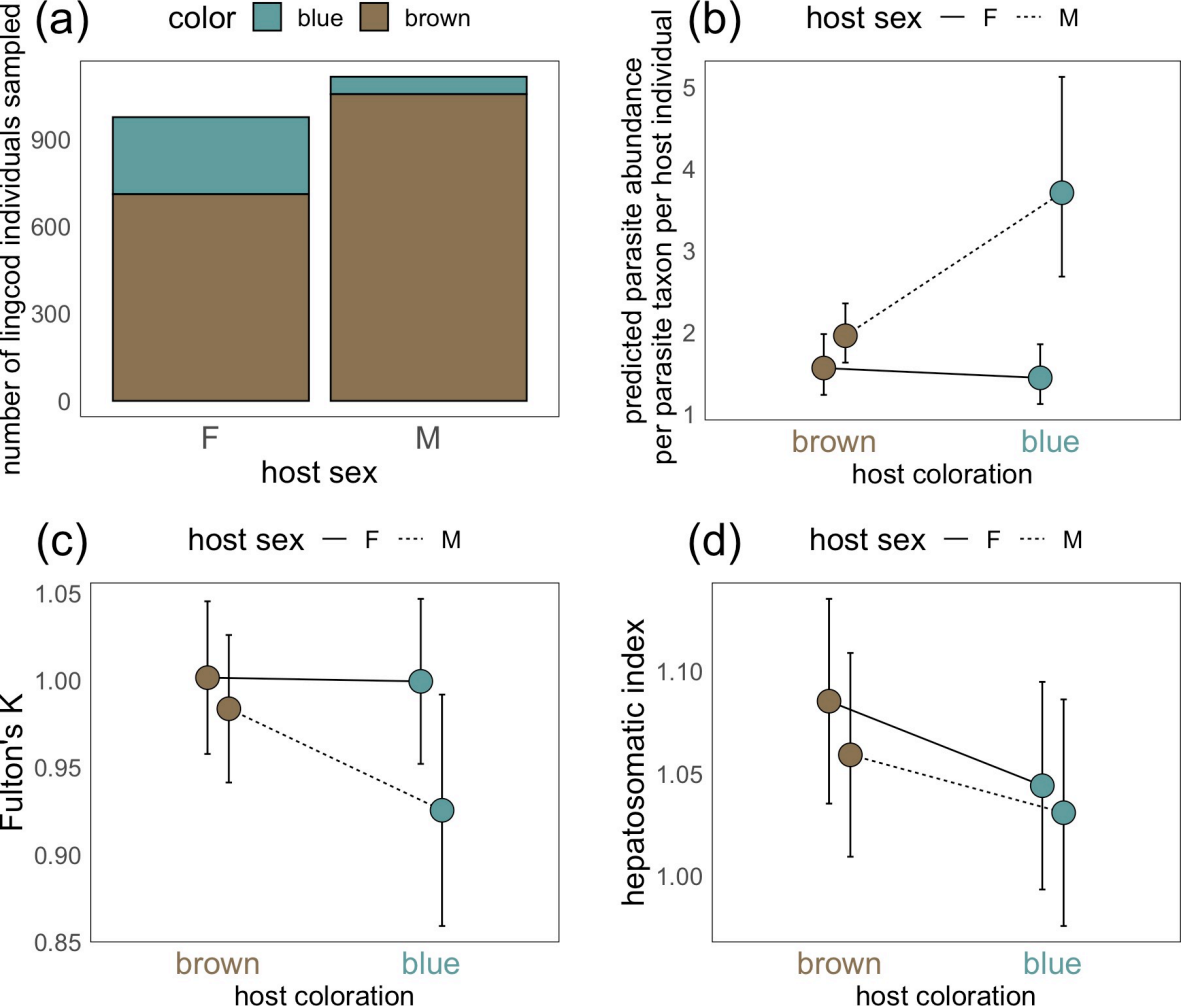
(a) Total number of lingcod individuals sampled of each sex (male versus female) and each color (blue versus brown). Effect of color (blue versus brown) and sex (male versus female) on (a) parasite abundance of the average parasite taxon per lingcod individual, (b) Fulton’s K condition factor (i.e., ratio of weight to cubed length), and (d) hepatosomatic index, where data represent predicted (fitted) values for the response of parasite abundance to color and sex, computed while keeping all other factors (including random effects) in the model constant using the *ggeffects()* function in the ggeffects package in R [44]. For (b), (c), and (d), points are slightly jittered in the x-dimension to allow visualization of overlapping error bars.

We collected a total of 2,251 lingcod, of which 2,091 were definitively identified as either male or female and as either blue or brown. The blue trait was observed more frequently among females (27.2% blue) than among males (5.4% blue; χ^2^ = 186.25, df = 1, p < 0.0001; **Fig 3A**). From among this initial group of lingcod collected, we analyzed the metazoan parasite burdens of 89 individuals, including 59 brown males, 4 blue males, 15 brown females, and 11 blue females. In these fish, we detected 29,465 parasite individuals across 25 taxa. A few parasite taxa dominated parasite detections. Specifically, larval trematodes (metacercariae) found in the fin and muscles of lingcod comprised 57% of all parasite detections. The next most abundant parasites were the adult trematode *Rhipidocotyle elongata* (9.8%), the larval acanthocephalan (cystacanth) *Corynosoma wegeneri* (5.8%), and copepod larvae (chalimus stage, 5.0%; **S1 Table**). The remainder of the parasite taxa we detected (21 taxa) each comprised fewer than 5% of all parasite detections and included copepods, isopods, monogeneans, trematodes, cestodes, nematodes, and acanthocephalans (**S1 Table**). Although these 21 taxa comprised only a small proportion of total detections, these parasites still reached high abundances in infected hosts; for example, each of the 36 lingcod infected with cestode larvae (plerocercoids) in the order Trypanorhyncha carried on average 33 plerocercoids, and the lingcod with the heaviest burden carried 358 plerocercoids.

Male lingcod carried 1.89 times more parasites if they were blue than if they were brown, whereas there was no difference in parasite burden between blue and brown female lingcod (**Table 1, Fig 3B**). Averaging across blue and brown individuals within each sex, male lingcod had 1.88 times more parasites than females (**Table 1**), but this difference was driven almost entirely by blue males; brown males and brown females carried similar parasite burdens (**Fig 3B**). Finally, lingcod collected from deeper depths tended to have lower parasite burdens than those collected from shallower depths (**Table 1**).

**Table 1:** Results of generalized linear mixed model for parasite burden across all parasite taxa.

Parameter	estimate	SE	z-value	p-value
depth	-0.11982	0.05441	-2.202	0.0276
sex[male]	0.94182	0.17238	5.464	< 0.0001
color[brown]	0.07987	0.13434	0.595	0.5522
sex[male]*color[brown]	-0.71694	0.17404	-4.119	< 0.0001

In this analysis, sample size (n) for individual fish hosts = 89, n parasite taxa = 25, n observations = 2225 (89 fish hosts * 25 parasite taxa), n sampling locations nested within sampling regions = 26, and n sampling regions = 4.

The permutation tests revealed that the significant interaction between color and sex was probably not due to random chance, despite the low sample size of blue males (n = 4). In the first iteration of the permutation test, in which color was randomly permuted only among male lingcod, the probability of obtaining a result as or more extreme (in either direction) than the actual result was 3.4%. In the second iteration of the permutation test, in which color was randomly permuted among both male and female lingcod, the probability of obtaining a result as or more extreme (in either direction) than the actual result was 5%.

**Fig 4:**
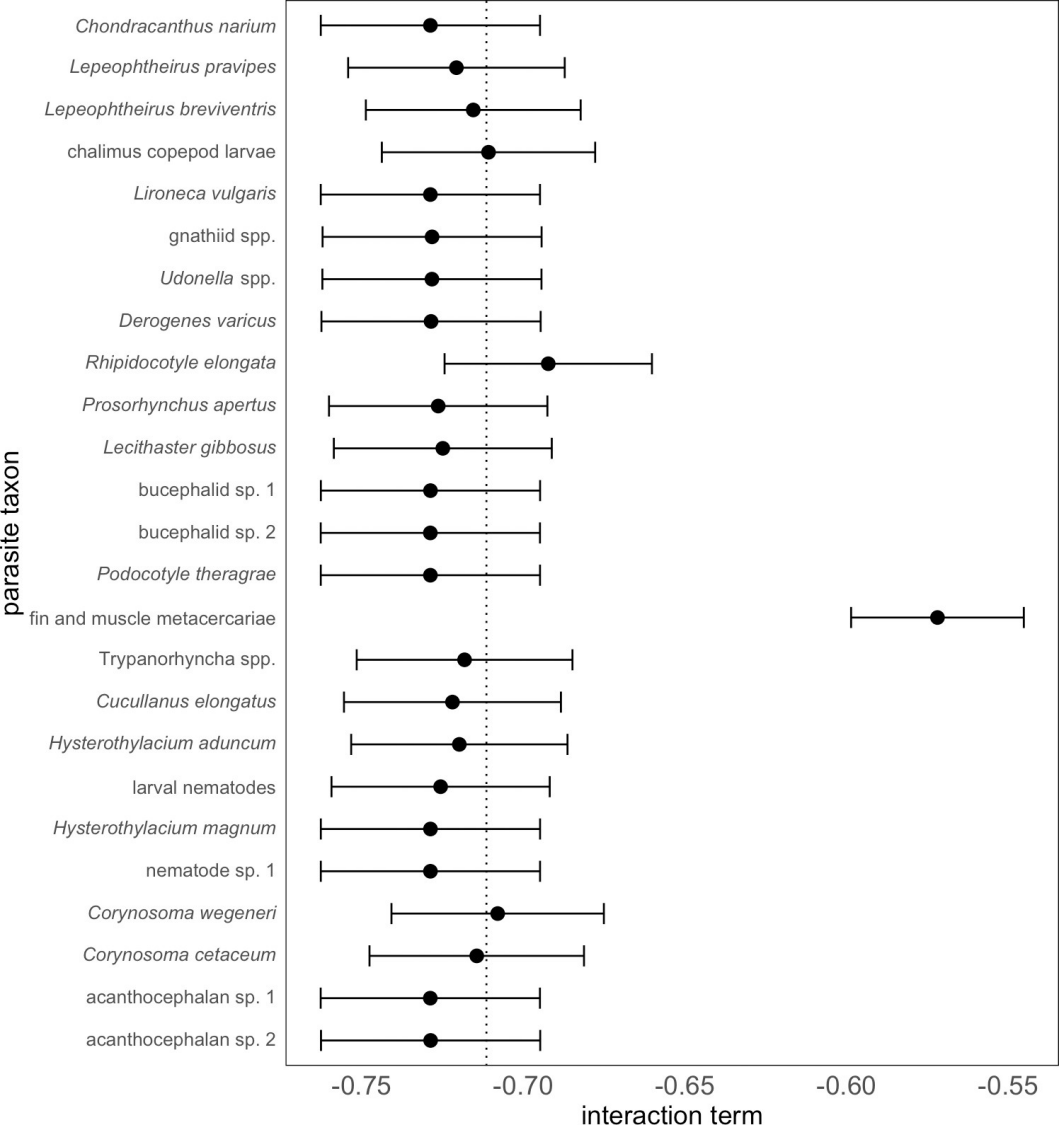
Random slopes for each parasite taxon’s response to the host color * sex interaction, from GLMM for parasite abundance. The across-parasite-taxa fixed effect of the interaction term is indicated with a dotted vertical line. A negative sex * color interaction indicates that the difference in parasite burden between blue and brown males is greater than the difference in parasite burden between blue and brown females; in other words, the more negative the interaction, the more parasites blue males carry compared to all other sex–color combinations.

The tendency for greater parasite abundance in blue males compared to all other sex–color combinations was consistent across all parasite taxa (i.e., all parasite taxa had similar responses to the sex * color interaction; **Fig 4**). In this analysis, a negative sex * color interaction indicates that the difference in parasite burden between blue and brown males is greater than the difference in parasite burden between blue and brown females; in other words, the more negative the interaction, the more parasites blue males carry compared to all other sex–color combinations. We extracted the random slopes for this interaction for each parasite taxon, and these are plotted in **Fig 4**, which shows that all parasite taxa were more abundant in blue males than in any other sex–color combination. Fin and muscle metacercariae had a weaker negative slope than any other parasite taxon, but that slope was still significantly different from zero, indicating that metacercariae are still more abundant in blue males than in any other sex–color combination, just to a lesser extent than other parasite taxa (**Fig 4**).

In alignment with the tendency for a consistent response to the sex * color interaction across parasite taxa, parasite community composition did not differ significantly among sex–color combinations, despite the higher total parasite burden in blue males (PERMANOVA; fixed effect of sex–color combination: F_3,59_ = 1.33, p = 0.1110, R^2^ = 0.04; **S1 Fig**). Although PERMANOVA revealed no significant differences in community composition among sex–color combinations, we note that blue and brown males tended to differ in their burdens of fin and muscle metacercariae, Trypanorhyncha spp. plerocercoids, *Corynosoma cetaceum* cystacanths, *Rhipidocotyle elongata* adult trematodes, and chalimus copepod larvae (**S1 Fig**).

Finally, our post-hoc analysis of Fulton’s K revealed a marginally non-significant interaction between color and sex, with a trend for blue male lingcod to have lower Fulton’s K (i.e., be skinnier for a given length) than lingcod of any other sex–color combination (**Table 2A; Fig 3C**). Our post-hoc analysis of the hepatosomatic index (HSI) revealed that, regardless of sex, blue lingcod had significantly lower HSI and hence reduced energy stores compared to brown lingcod (**Table 2B; Fig 3D**). HSI increased with increasing depth (**Table 2B**).

**Table 2:** Results of general linear mixed models for (a) Fulton’s K and (b) hepatosomatic index. (a) Fulton’s K (n unique fish hosts = 2087, n sampling locations nested within sampling regions = 87, and n sampling regions = 7). (b) hepatosomatic index (n unique fish hosts = 2076, n sampling locations nested within sampling regions = 87, and n sampling regions = 7).

(a)				
**parameter**	**estimate**	**SE**	**z-value**	**p-value**
depth	-0.0163	0.0101	-1.61	0.1070
sex[male]	-0.0739	0.0295	-2.50	0.0124
color[brown]	0.0021	0.0169	0.13	0.8990
sex[male]*color[brown]	0.0561	0.0320	1.75	0.0799
(b)				
depth	0.0207	0.0076	2.74	0.0061
sex[male]	-0.0132	0.0140	-0.94	0.3454
color[brown]	0.0413	0.0081	5.10	<0.0001
sex[male]*color[brown]	-0.0130	0.0152	-0.86	0.3919

## Discussion

Sexual dimorphism in color hints at fundamental ecological or evolutionary factors that underpin a species’ dynamics. The results presented here showed that blue coloration was more common in female lingcod, but only in male lingcod was blue coloration associated with elevated parasite burden. This pattern could arise due to parasite-induced physiological damage, or high parasite burdens and blueness may both be driven by the same physiological mechanism (e.g., starvation). Below, we explore the elements of each mechanism that are supported (or not supported) by our data.

Blue males were encountered much less frequently than other sex–color combinations, resulting in a low sample size of that color morph. Despite this, the data suggest that blue male lingcod are characterized by heavier parasite burdens than other color–sex combinations in this species. The four highly parasitized blue males that drive this significant interaction were collected from geographically distant sites (**Fig 1**), so color is not confounded with geography, and blue males were collected across a broad subset (10.7–36.9 m) of the total depth range (7.6–72.2 m). These features of the data set imply that the pattern of high rates of parasitism among blue males is not an artifact of our sampling strategy and instead reflects a natural phenomenon.

Our data do not support the hypothesis that blueness is a sexually selected trait. If blue coloration were an “honest signal” of parasite resistance, then we would expect lower parasite burdens in blue individuals, which is the opposite of what we observed. Blue coloration could be an “honest signal” of parasite tolerance, since we did observe higher parasite burdens among blue males than among brown males; this interpretation requires us to assume that blue coloration is a trait that is sexually selected in both male and female lingcod, since blue coloration was found in both females and males, and was in fact more common among females. Perhaps blue coloration signals parasite tolerance, but only in males, who already have an immune disadvantage that compounds the resistance-eroding effects of blueness. However, the distribution of blueness across the sexes and the lack of a difference in parasite burden between blue and brown female lingcod argue against a role for the “immunocompetence handicap hypothesis”.

Our data are also inconsistent with the hypothesis that blue coloration is an incidental outcome of parasitic infection, because we had originally assumed that parasitic infection would be equally physiologically disruptive for male and female lingcod, and that the sexes would therefore exhibit equal differences in burden between blue and brown individuals. However, if one sex (e.g., females) is likelier to die of parasitic infection, we would expect to see a smaller difference in burden between blue and brown individuals for the sex with the greater vulnerability. Alternatively, if the blue trait is initiated at different parasite thresholds between the sexes, we would expect to see a smaller difference in burden between blue and brown individuals for the sex with the lower threshold. If the parasite burden threshold that initiates blue coloration in females is very small, then it may not be detected in our study (**Fig 3B**). Other examples of divergence between sexes in sensitivity to the pathological effects of parasitism exist; for instance, among honey bees (*Apis mellifera*) experimentally exposed to the gut parasite *Nosema ceranae*, males grow less and die sooner than females [45]. However, we expected that, if this hypothesis were supported, one or a few parasite species (perhaps those associated with the liver or gallbladder) would drive the difference in parasite burden between blue and brown individuals; instead, we saw that all parasites contributed to the interaction of sex and color (**Fig 4**).

We cannot rule out the possibility that both blue coloration and parasite burden are driven by some unmeasured variable. In order to explain our observations, this variable would need to (1) make blue coloration more common in females and (2) drive a correlation between color and parasite burden in only males. Perhaps blue males are particularly likely to die of their heavy parasite burdens, making blue coloration appear more common in females. Alternatively, perhaps blue coloration is a reflection of diet differences between blue and brown lingcod (as hypothesized by Galloway et al. [18]) and those diet differences produce an elevated parasite burden only in blue males due to the inherent susceptibility of males to infection [19, 20]. On one hand, if this were the case, we would not expect an equal sex * color interaction across most parasites, because not all parasites are transmitted to lingcod via food (**S1 Table**). On the other hand, perhaps males spend more time at depths or in habitats that expose them to both directly and trophically transmitted parasites [46] and blue males are exceptionally susceptible to being infected by these parasites. These possibilities are consistent with lingcod ecology: male lingcod tend to occupy habitats at shallower depths than females [46], which may dictate sex-specific differences in available prey, and males remain sedentary for long periods while guarding nests, which may lead to sex-specific differences in direct exposure to parasites. Alternatively, perhaps blue coloration reflects the results of infection by a non-metazoan parasite that interferes with immunity against parasitic infection. For example, perhaps a liver- or gallbladder-associated virus, bacterium, protozoan, or myxozoan damages the organ, releases biliverdin, and is more common among female lingcod, but only compromises the ability of males to resist parasitic infection due to males’ inherent pre-existing susceptibility to infection [19, 20]. Finally, perhaps blue coloration reflects exposure to stressors such as starvation, and only compromises the ability of males to resist parasitic infection due to males’ inherent pre-existing susceptibility to infection [19, 20]. None of these possibilities linking unmeasured variables to coloration and parasite burden can be ruled out by our data.

Our post-hoc analysis of lingcod body condition (**Table 2, Fig 3C**) suggests that low body condition is associated with blueness. We measured lower hepatosomatic index (HSI) values among blue fish than brown fish regardless of sex. These data suggest that low body condition (e.g., starvation) could cause blueness and, for male individuals whose immune systems are already compromised by male sex hormones, that low body condition may also increase susceptibility to parasitic infection–a conclusion that is supported by Galloway et al. [18], who found that blue individuals are, on average, smaller (lower total length) than brown individuals. Additionally, we found that blue and brown female lingcod had statistically indistinguishable Fulton’s K values, whereas blue male lingcod had a marginally non-significant trend toward lower Fulton’s K values (p = 0.08) than brown male lingcod. This finding suggests either that those males already compromised by their low body condition are exceptionally likely to acquire high parasite burdens, or that the high parasite burdens experienced by blue males further erode their body condition relative to blue female conspecifics. Of course, this study is correlational, and it is therefore equally possible that low body condition causes blueness, that blueness causes low body condition, or that both are caused by some third factor. Future experiments in which lingcod are placed on restricted diets will reveal whether low body condition leads to blueness.

The fact that all parasite taxa contributed to the difference between blue and brown males (**Fig 4**) suggests that whatever mechanism is at play affects most metazoan parasites equally. Although the response of fin and muscle metacercariae to the sex * color interaction was weaker than the response of the remainder of the parasites, it was nonetheless significantly negative, indicating that blue males carried a heavier burden of fin and muscle metacercariae than did brown males, blue females, and brown females. Therefore, whatever drives this effect acts in the same direction on all of the metazoan parasite taxa we detected. This strongly points to the immune system as a driver, and further supports the hypotheses above linking blue coloration with reduced immune efficacy in males.

The role of the immune system in the association between blueness and parasitism is also supported by findings from a parallel study conducted on lingcod collected during the same sampling campaign [18]. Galloway et al. [18] screened for differences in the fatty acid signatures of fish across both colors and sexes, finding that blue individuals possessed lower concentrations of ω-6 fatty acids–precursor molecules for anti-inflammatory eicosanoids, but finding no differences in fatty acids between the sexes. If blueness is associated with reduced immune capacity, as Galloway et al.’s work suggests, this would be especially detrimental for male lingcod, which are already at an immunological disadvantage [19, 20]. Galloway et al.’s work [18] corroborates color-associated differences in an important component of the fish immune system.

That a much greater proportion of female than male lingcod were blue suggests that different factors may be shaping the expression of the blue trait in lingcod males versus females. It is possible that the blue trait is sexually selected in both male and female lingcod, and that males are the choosier sex, driving a greater proportion of females to evolve or express blue coloration. This is compatible with the unusual mating system of lingcod, in which lingcod males exhibit nest-tending behavior, attracting females to a territory and then fertilizing and guarding the eggs laid there, whereas females invest little in parental care after egg laying [21]. Alternatively, the blue trait might have nothing to do with sexual selection, and might instead arise due to diet, infection with a non-metazoan parasite (e.g., a virus or bacterium), starvation, or some other confounding factor that is more likely to affect female than male lingcod (see above). If, for example, starvation is involved in blueness and females are more likely to starve, we would expect a greater frequency of the blue trait among females. Our data are also consistent with the interpretation that female lingcod start expressing the blue trait at lesser degrees of starvation, but male lingcod need to be especially compromised to express blueness.

The mystery of why lingcod are blue remains, but this study reveals some interesting clues. Our data suggest that immunocompetence may be associated with the blue trait, since (1) the effects we observed were consistent across all of the metazoan parasites we detected, and ecological drivers of the blue versus brown difference are unlikely to have consistent effects across such a great diversity of parasite taxa, and (2) effects were observable only in males, the sex that tends to have inherently lower immunocompetence across the vertebrates. It is possible that parasites induce physiological damage that produces blueness or that blue coloration and parasite burden may be driven by some shared physiological mechanisms, such as starvation. Although our study cannot discriminate among these possibilities, our data suggest the involvement of the immune system in the blue lingcod polymorphism–an exciting jumping-off point for future research to definitively identify the cause of lingcod blueness and a hint that immunocompetence and parasitism may play a role in lingcod population dynamics.

## Supporting information

S1 Fig Results of PERMANOVA. (DOCX)

S1 Table Parasite taxa detected in lingcod. (DOCX)

S1 Text Detailed methods for parasitological dissections. (DOCX)
