# Supplementary material for: The weaker sex: Male lingcod (Ophiodon elongatus) with blue color polymorphism are more burdened by parasites than are other sex–color combinations
Source: PLoS One. 2021 Dec 31;16(12):e0261202. doi: 10.1371/journal.pone.0261202 (PMC8719767; doi:10.1371/journal.pone.0261202)
Supplement: S1 Fig — (DOCX) [file pone.0261202.s001.docx]

**
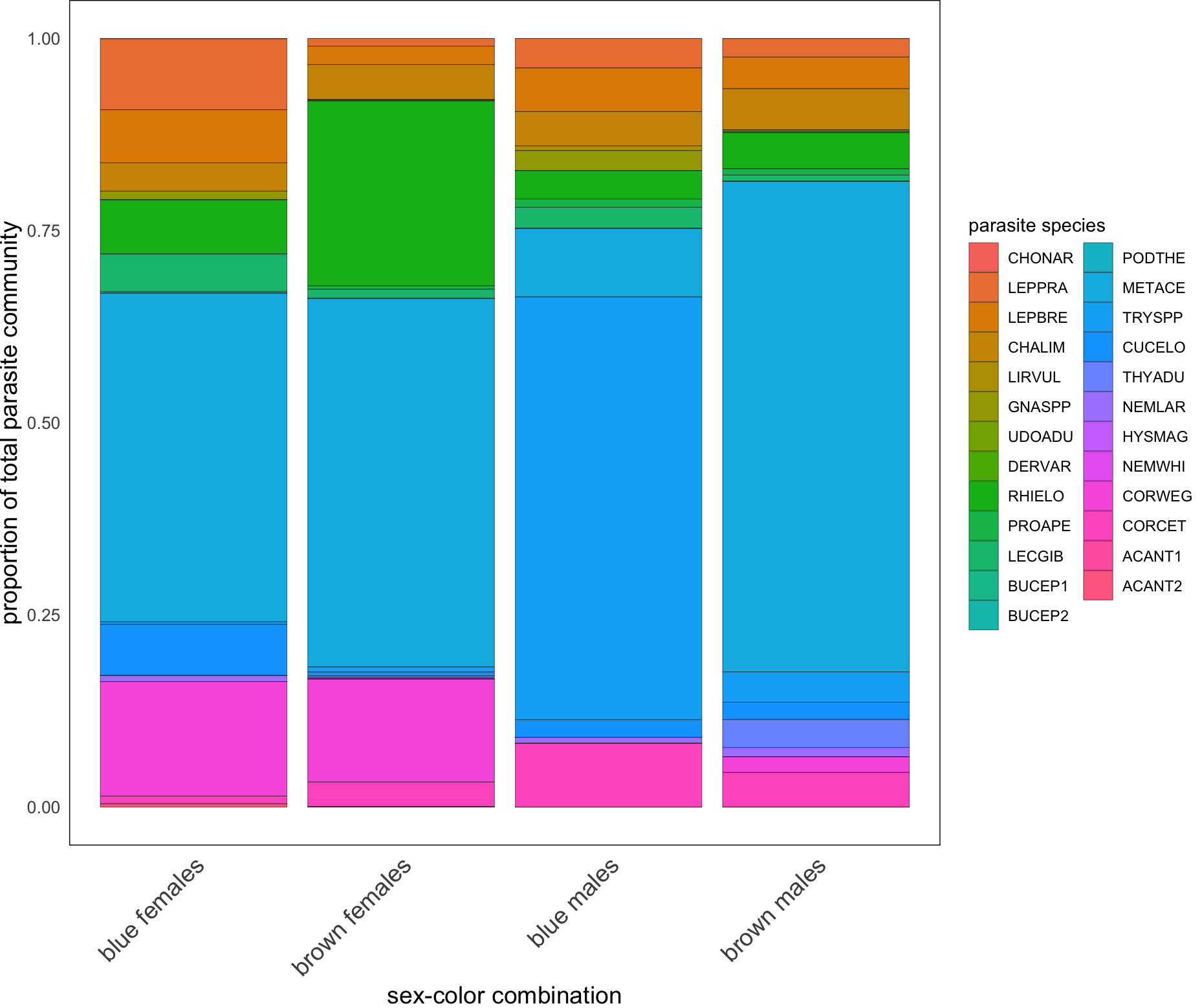
**

**Supporting Information Figure 1.** PERMANOVA revealed no significant differences (p = 0.11) in community composition among sex–color combinations, despite there being higher total parasite burdens in blue male lingcod. Plot shows the proportion of the total parasite community comprised of each parasite species for all sex–color combinations of lingcod. Parasite taxon codes are the first three letters of genus and species names (displayed in order in **Figure 4**) and are linked to full taxon names in **Supporting Information Table 2**.
