# Supplementary material for: The weaker sex: Male lingcod (Ophiodon elongatus) with blue color polymorphism are more burdened by parasites than are other sex–color combinations
Source: PLoS One. 2021 Dec 31;16(12):e0261202. doi: 10.1371/journal.pone.0261202 (PMC8719767; doi:10.1371/journal.pone.0261202)
Supplement: S1 Table — (DOCX) [file pone.0261202.s002.docx]

**Supporting Information Table 1.** Parasite taxa detected in lingcod (*Ophiodon elongatus*). Taxonomic grouping = broader taxonomic group to which each parasite taxon belongs. Life stage = whether the parasite was found in lingcod in its adult or larval form. Mode of transmission to lingcod = the life stage that is infectious to lingcod and gives rise to the life stage observed in lingcod. Total abundance = total number of parasite individuals of this parasite taxon counted across all 89 lingcod host individuals. % of total abundance = total number of parasite individuals of this parasite taxon / total number of parasite individuals of all parasite taxa.

| **Taxonomic grouping** | **Parasite taxon** | **Taxon code** | **Life stage** | **Mode of transmission to lingcod** | **Total abundance** | **% of total abundance** |
| --- | --- | --- | --- | --- | --- | --- |
| Copepoda | *Chondracanthus narium* | CHONAR | adult | host-seeking pelagic larva | 4 | 0.01 |
|  | *Lepeophtheirus pravipes* | LEPPRA | adult | host-seeking pelagic larva | 758 | 2.57 |
|  | *Lepeophtheirus breviventris* | LEPBRE | adult | host-seeking pelagic larva | 1,151 | 3.91 |
|  | chalimus copepod larvae | CHALIM | larva (chalimus) | host-seeking pelagic larva | 1,474 | 5.00 |
| Isopoda | *Lironeca vulgaris* | LIRVUL | adult | host-seeking pelagic larva | 6 | 0.02 |
|  | gnathiid spp. | GNASPP | adult | host-seeking benthic larva | 72 | 0.24 |
| Monogenea | *Udonella* spp. | UDOADU | adult | host-seeking pelagic larva | 39 | 0.13 |
| Trematoda | *Derogenes varicus* | DERVAR | adult | trophically transmitted | 19 | 0.06 |
|  | *Rhipidocotyle elongata* | RHIELO | adult | trophically transmitted | 2,876 | 9.76 |
|  | *Prosorhynchus apertus* | PROAPE | adult | trophically transmitted | 195 | 0.66 |
|  | *Lecithaster gibbosus* | LECGIB | adult | trophically transmitted | 363 | 1.23 |
|  | bucephalid sp. 1 | BUCEP1 | adult | trophically transmitted | 8 | 0.03 |
|  | bucephalid sp. 2 | BUCEP2 | adult | trophically transmitted | 2 | 0.01 |
|  | *Podocotyle theragrae* | PODTHE | adult | trophically transmitted | 3 | 0.01 |
|  | fin and muscle metacercariae | METACE | larva (metacercaria) | host-seeking pelagic larva | 16,811 | 57.05 |
| Cestoda | Trypanorhyncha spp. | TRYSPP | larva (plerocercoid) | trophically transmitted | 1,171 | 3.97 |
| Nematoda | *Cucullanus elongatus* | CUCELO | adult | trophically transmitted | 627 | 2.13 |
|  | *Hysterothylacium aduncum* | THYADU | adult | trophically transmitted | 715 | 2.43 |
|  | larval nematodes | NEMLAR | larva | trophically transmitted | 264 | 0.90 |
|  | *Hysterothylacium magnum* | HYSMAG | adult | trophically transmitted | 4 | 0.01 |
|  | nematode sp. 1 | NEMWHI | adult | trophically transmitted | 4 | 0.01 |
| Acantho-  cephala | *Corynosoma wegeneri* | CORWEG | larva (cystacanth) | trophically transmitted | 1,705 | 5.79 |
|  | *Corynosoma cetaceum* | CORCET | larva (cystacanth) | trophically transmitted | 1,180 | 4.00 |
|  | acanthocephalan sp. 1 | ACANT1 | adult | trophically transmitted | 1 | 0.003 |
|  | acanthocephalan sp. 2 | ACANT2 | adult | trophically transmitted | 13 | 0.04 |
