# Supplementary material for: The weaker sex: Male lingcod (Ophiodon elongatus) with blue color polymorphism are more burdened by parasites than are other sex–color combinations
Source: PLoS One. 2021 Dec 31;16(12):e0261202. doi: 10.1371/journal.pone.0261202 (PMC8719767; doi:10.1371/journal.pone.0261202)
Supplement: S1 Text — (DOCX) [file pone.0261202.s003.docx]

**Supporting Information Text 1.** Detailed methods for parasitological dissections.

For each of the 89 fish selected for parasitological dissection, we performed a comprehensive parasitological examination designed to detect most metazoan parasites. First, the skin and buccal cavity were searched for ectoparasites. Then, we removed the right pectoral, right pelvic, anal, dorsal, and caudal fins and examined them with transmitted light under a stereomicroscope to detect trematode metacercariae. We removed the right gills, right eye, right skin and filet, and viscera and examined the body cavity. The surface of the liver was thoroughly examined. All other internal organs were squashed between glass plates and examined with transmitted light under a stereomicroscope. We cut the gill arches apart from one another, shook them in a jar of seawater to dislodge mucus, and then examined the entire gill arch and the wash under magnification. The right filet was removed, squashed between glass plates, and examined with transmitted light under a stereomicroscope. The inner surface of the skin removed from the fillet was examined. Finally, we examined the water in the bottom of the bag in which each individual fish was stored to count any dislodged parasites. For some bilateral organs, only one side was examined (i.e., eyeball, pectoral fin, pelvic fin, filet, skin, gills). Therefore, we doubled the count of any parasite found in these organs to create our final tally of parasites. For ectoparasites, only one side of the body was scanned for parasites, but it is possible that parasites from the other side were washed into the "flush" (i.e., the water in the bottom of the bag). To deal with this, we doubled the number of ectoparasites found on bilateral external organs (e.g., skin), and added the number of ectoparasites found in the flush. We archived photographs of each parasite taxon and created an illustrated and annotated identification guide for use by dissectors, to maximize consistency across the entire period of dissection (2 Feb 2017–14 May 2019). We also retained voucher specimens for each parasite taxon (available by request to the corresponding author).
